# Efficacy and safety of telitacicept in adults with systemic lupus erythematosus: a systematic review and stratified meta-analysis

**DOI:** 10.3389/fmed.2026.1959776

**Published:** 2026-09-25

**Authors:** Zihua Wang, Huan Li, Jigao Li, Wen Fu, Songwei Li, Fuhai Feng, Dezhen Wang, Peipei Su

**Affiliations:** Department of Rheumatology, The First Affiliated Hospital of Henan University of Chinese Medicine, Zhengzhou, China

**Keywords:** B-cell-targeted therapy, lupus nephritis, systematic review and meta-analysis, systemic lupus erythematosus, telitacicept

## Abstract

**Background:**

Telitacicept is a TACI-Fc fusion protein that targets both B-cell activating factor (BAFF) and a proliferation-inducing ligand (APRIL). Evidence in adults with systemic lupus erythematosus (SLE) includes placebo-controlled randomized trials, other comparative studies, and single-arm cohorts.

**Objectives:**

To assess the efficacy and safety of telitacicept in adults with SLE and to synthesize placebo-controlled randomized trials, other comparative studies, and single-arm treatment cohorts separately by study design.

**Methods:**

We searched electronic databases, screened references, and conducted hand searches, and cross-checked international trial registries. Eligible studies enrolled adults with SLE, including lupus nephritis (LN) subgroups. Comparative binary outcomes were pooled as risk ratios (RRs), continuous outcomes as mean differences (MDs), and single-arm outcomes as logit-transformed proportions. Random-effects models were used, and evidence certainty was assessed using GRADE.

**Results:**

Thirty-nine reports, all from China, were included, and 22 contributed to quantitative synthesis. In two placebo-controlled randomized trials, telitacicept 160 mg plus standard therapy increased SLE Responder Index-4 (SRI-4) response at 48–52 weeks (RR = 2.04, 95% CI 1.66–2.50; I2 = 0.0%) and slightly increased any adverse events (RR = 1.09, 95% CI 1.02–1.17). No clear difference was observed for serious adverse events (RR = 0.68, 95% CI 0.35–1.30). Observational comparative findings in adult LN and single-arm cohort proportions were assessed separately. GRADE rated SRI-4 response and any adverse event as moderate certainty, serious adverse events as low certainty, and all other outcomes as very low certainty.

**Conclusion:**

In the Chinese populations studied, telitacicept 160 mg plus standard therapy probably improves SRI-4 response at 48–52 weeks in adults with active SLE and may slightly increase any adverse events. Evidence for other outcomes is uncertain and does not establish comparative efficacy or long-term safety.

**Systematic Review Registration:**

https://www.crd.york.ac.uk/PROSPERO/view/CRD420261464193, PROSPERO CRD420261464193.

## Introduction

Systemic lupus erythematosus (SLE) is a heterogeneous autoimmune disease characterized by loss of immune tolerance, autoantibody formation, and inflammatory injury in multiple organs (1). Current treatment goals are to control disease activity, prevent irreversible organ damage, reduce glucocorticoid exposure, and achieve remission or lupus low disease activity state (LLDAS). Despite the widespread use of antimalarials, glucocorticoids, immunosuppressants, and biologic agents, some patients experience persistent activity, relapse, treatment toxicity, or organ-threatening disease (2).

Telitacicept is a transmembrane activator and calcium-modulator and cyclophilin ligand interactor (TACI)-Fc fusion protein that simultaneously targets B-cell activating factor (BAFF, also known as BLyS) and a proliferation-inducing ligand (APRIL). It consists of the extracellular domain of TACI fused to the Fc portion of human immunoglobulin G (3, 4). Unlike single-target biologic agents that selectively inhibit soluble BAFF/BLyS, such as belimumab, telitacicept is a recombinant fusion protein that binds both BAFF/BLyS and APRIL. BAFF primarily supports the survival and differentiation of transitional and mature B cells, whereas APRIL is associated with the survival of plasmablasts and long-lived plasma cells (5, 6). Dual-target blockade may therefore affect B-cell maturation, plasma-cell survival, and immunoglobulin production, but its specific effects and clinical benefit in SLE require clinical validation (7).

Clinical trials have evaluated the efficacy and safety of telitacicept in adults with active SLE. Subsequent real-world studies have extended the evidence base to comparisons with conventional therapy or belimumab, lupus nephritis (LN), and treatment-target attainment. However, the stringent eligibility criteria of randomized controlled trials (RCTs) may not fully represent complex routine practice, including patients with refractory organ involvement, multiple comorbidities, or failure of several previous immunosuppressive therapies. Recent real-world evidence (RWE) has added to understanding of telitacicept in more heterogeneous populations, although most studies are small and methodologically heterogeneous.

No evidence synthesis has rigorously distinguished core RCT evidence from the real-world evidence base while stratifying efficacy, glucocorticoid-sparing effects, and safety. Placebo-controlled randomized trials, other comparative studies, and single-arm cohorts afford different strengths of causal inference. They also differ in comparators, disease phenotypes, response definitions, and follow-up duration. Pooling these designs into a single overall effect could obscure design-specific bias and increase the risk of duplicate inclusion of patients or outcomes. We therefore conducted a stratified systematic review and meta-analysis. Evidence for SRI-4 and safety from placebo-controlled randomized trials was considered primary, whereas other comparative studies and single-arm treatment cohorts were pooled separately. Pooled single-arm findings were interpreted only as event proportions in telitacicept-treated cohorts.

## Methods

### Protocol and reporting standard

This systematic review was designed and reported in accordance with the Preferred Reporting Items for Systematic Reviews and Meta-Analyses 2020 statement (PRISMA 2020) (8). The protocol was registered in PROSPERO (CRD420261464193).

### Study design and eligibility criteria

Eligibility criteria were prespecified according to PICOS. Eligible participants were adults aged 18 years or older with SLE diagnosed according to accepted classification or diagnostic criteria. Adult LN was a prespecified disease subgroup. Pediatric studies and mixed-age studies without separable adult data were excluded. The intervention was subcutaneous telitacicept, usually administered with standard therapy. Comparators could include placebo plus standard therapy, conventional therapy, standard therapy alone, or another biologic agent. Placebo-controlled randomized trials, other comparative studies, and adult single-arm cohorts entered their respective models. Eligible reports were full peer-reviewed articles and had to provide at least one prespecified efficacy or safety outcome as an event count, denominator, mean, or measure of dispersion. Outcomes of interest included SRI-4, disease activity, LLDAS or remission, renal response, proteinuria, complement levels, glucocorticoid exposure, adverse events, and infection. Case reports, case series, conference abstracts, reviews, guidelines, commentaries, research protocols, economic evaluations, and duplicate publications were excluded from the main quantitative syntheses. When reports described the same or potentially overlapping cohort, only the report with the most complete sample, extractable outcomes, or adjusted analysis was retained for a given model.

### Information sources and search strategy

We searched PubMed/MEDLINE, the Cochrane Library/CENTRAL, China National Knowledge Infrastructure (CNKI), Wanfang Data, and the VIP Database. We also screened the references of included studies and conducted hand searches. Searches covered database inception through 30 July 2026. Supplementary searches of ClinicalTrials.gov and the World Health Organization International Clinical Trials Registry Platform (WHO ICTRP), including ChiCTR records cross-checked through WHO ICTRP, covered registry inception through 30 July 2026 and used telitacicept, RC18, and TACI-Ig combined with lupus-related terms. These searches identified trial registrations but no additional eligible adult SLE study with publicly available full results or eligible full report from outside China. Registry records were used only to assess missing evidence and were not added to the PRISMA record count. Embase, Web of Science, and SinoMed were not searched because access was unavailable. The core PubMed search strategy was: ("Lupus Erythematosus, Systemic"[MeSH Terms] OR "Systemic Lupus Erythematosus"[Title/Abstract] OR "SLE"[Title/Abstract] OR "Lupus"[Title/Abstract]) AND ("Telitacicept"[Supplementary Concept] OR "Telitacicept"[Title/Abstract] OR "RC18"[Title/Abstract]).

### Study selection and data extraction

Titles, abstracts, and full texts were assessed against the eligibility criteria. Data were entered into a standardized workbook and verified item by item against original PDF pages, tables, or figures. Extracted data included study design, population, interventions and comparators, outcome definitions, follow-up duration, event counts or means, denominators, and measures of dispersion. For duplicate or potentially related cohorts, only one report was retained for each comparable model, or a prespecified alternative-report sensitivity analysis was conducted. Potential duplicate or overlapping cohorts were assessed using study centers, recruitment periods, intervention and comparator characteristics, sample sizes, baseline characteristics, follow-up durations, and outcome time points. For each outcome model, the report providing the most complete sample, most comprehensive extractable data, or most appropriate adjusted analysis was selected. Potentially overlapping reports were evaluated through alternative-report sensitivity analyses, and any unresolved partial overlap was considered when interpreting the findings.

### Outcomes

The primary efficacy outcome was SLE Responder Index-4 (SRI-4) response at 48–52 weeks in placebo-controlled randomized trials. Primary safety outcomes were any adverse event, serious adverse event, and upper respiratory tract infection. Secondary comparative outcomes included clinical total effective response, the Systemic Lupus Erythematosus Disease Activity Index (SLEDAI) or SLEDAI-2000 (SLEDAI-2 K), complement C3 and C4, infection, complete renal response (CRR), and primary efficacy renal response (PERR). Single-arm outcomes included SRI-4, LLDAS, CRR, any adverse event, and infection at different time points. Different response definitions and follow-up windows were not combined into a single overall effect.

### Risk of bias, certainty of evidence, and missing evidence

For randomized trials, the Cochrane Risk of Bias 2 tool (RoB 2) (9) was applied by outcome to assess randomization, deviations from intended interventions, missing outcome data, outcome measurement, and selective reporting, with an overall judgment assigned. The Newcastle-Ottawa Scale (NOS) (10) was used for comparative observational studies, and the JBI Critical Appraisal Checklist for Case Series (11) was used for uncontrolled treatment cohorts. The JBI checklist was used only for methodological appraisal and did not change the study-design classification. Two reviewers independently completed all assessments. Disagreements were resolved through discussion and, when necessary, adjudication by a third reviewer. Certainty of evidence for important outcomes was assessed using the Grading of Recommendations Assessment, Development and Evaluation (GRADE) approach (12). Randomized trials started at high certainty, whereas observational comparative and single-arm descriptive evidence started at low certainty. Certainty was downgraded for risk of bias, inconsistency, indirectness, imprecision, and publication bias, and upgrading factors were also considered. For single-arm models, GRADE applied only to the descriptive certainty of proportions in treated cohorts and not to treatment-effect inference. Funnel plots and regression-based asymmetry tests, including Egger's test, were not performed for models with fewer than 10 studies. The risk of missing evidence was additionally considered by comparing published reports with the supplemental registry search.

### Statistical analysis

Comparative binary outcomes were pooled as risk ratios (RRs), continuous outcomes as mean differences (MDs), and single-arm binary outcomes as proportions after logit transformation, all with 95% confidence intervals (CIs). The logit transformation was selected because it maps proportions to an unconstrained scale for random-effects modeling while constraining back-transformed pooled and predicted proportions to the 0–1 range; it also accommodates conventional continuity correction for zero-event cells and remains clinically interpretable after back-transformation. We did not use the Freeman-Tukey double-arcsine transformation because its back-transformed pooled proportions can be difficult to interpret clinically and its weighting and back-transformation behavior can be less transparent with heterogeneous, unevenly sized cohorts. Primary analyses used random-effects models estimated by restricted maximum likelihood. Hartung-Knapp adjustment was applied when at least three studies were available. With only two studies, random-effects CIs based on the z distribution were retained. Fixed-effect estimates were used as sensitivity analyses. Heterogeneity was described using *τ*^2^, Cochran's Q, and I^2^. Prediction intervals and leave-one-out analyses were calculated when at least three studies were available. Sensitivity analyses examined telitacicept dose, study-design limitations, replacement of potentially overlapping reports, follow-up duration, and limitations in denominator information. Analyses were performed using R version 4.6.0.

## Results

### Study selection and analytic dataset

We identified 361 records, including 355 from databases (PubMed/MEDLINE, 102; Cochrane Library/CENTRAL, 22; CNKI, 68; Wanfang Data, 128; and VIP, 35) and six from reference screening and hand searches. After 143 duplicates were removed, 218 titles and abstracts were screened and 167 were excluded. We sought 51 full texts, of which six could not be retrieved, leaving 45 for full-text assessment. Of the 45 reports assessed for eligibility, six were excluded after full-text assessment: one because of internally inconsistent data, three because adult SLE data could not be separately extracted, and two because they were duplicate reports or involved overlapping cohorts. Ultimately, 39 study reports were included, of which 22 contributed to at least one quantitative meta-analysis model (Figure 1).

**Figure 1:**
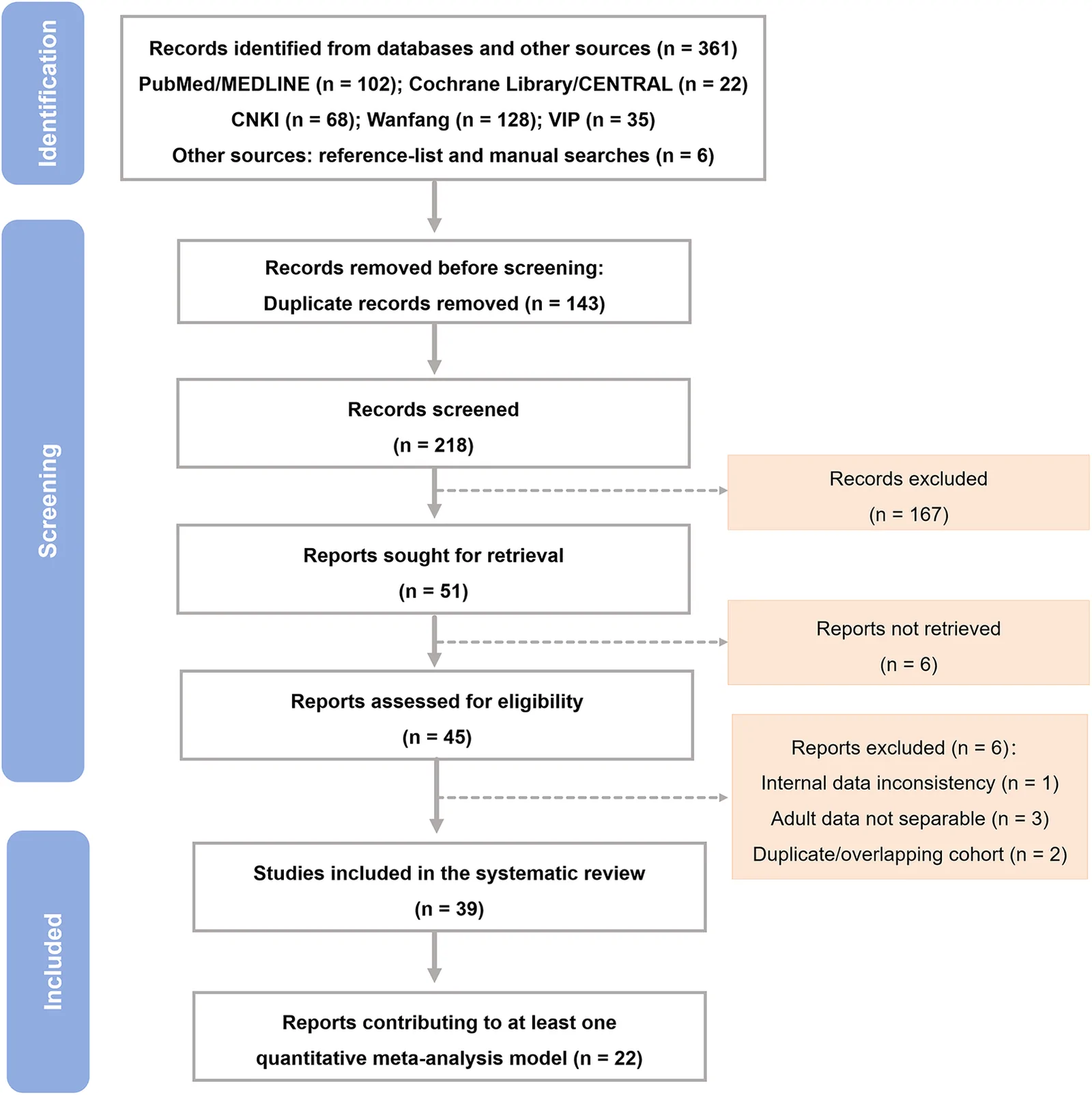
PRISMA 2020 flow diagram for study identification, screening, eligibility assessment, and inclusion. Searches covered database inception through 30 July 2026.

### Study characteristics and risk of bias

The 39 included reports comprised two placebo-controlled randomized trials, studies comparing telitacicept with conventional therapy or belimumab, comparative adult LN cohorts, and prospective or retrospective single-arm studies. All studies were conducted in China, and most nonrandomized studies were single-center or small multicenter cohorts. No eligible full report from outside China was identified through the searched bibliographic sources or supplemental trial-registry search. Table 1 presents the characteristics of included studies. Table 2 summarizes the evidence modules, quantitative models, number of studies, and sample sizes. Table 3 lists the identifier, outcome, and effect estimate for each model.

**Table 1:** Basic characteristics of the included studies.

Author, year	Sample size, n	Age, years	Follow-up	Study design	Outcome codes
Yang, 2026 (13)	63	33.16 ± 6.87/33.19 ± 6.85	12 mo	Comparative clinical study	1,5,14,15,17
Jia, 2026 (14)	40	47.85 ± 6.37/48.54 ± 6.73	NR	Non-randomized dose-comparison study	1,5,7,9,10,14,15,17
Sun et al., 2026 (15)	20	42.50 ± 13.15	12 mo	Prospective observational cohort	3,15-17,20
Wang et al., 2026 (16)	40	34.40 ± 14.96/31.15 ± 10.81	12 mo	Retrospective comparative LN cohort	6,15
Wei et al., 2026 (17)	94	33.26 ± 11.76	Up to 36 mo	Retrospective single-arm cohort	1-3,7-18
Zhang et al., 2026 (18)	21	38.38 ± 11.81	≥12 mo; median 24 mo	Retrospective single-arm LN cohort	6-13,15,16,18
Zhang et al., 2025a (19)	82	31.74 ± 7.28/31.07 ± 6.76	24 wk	Comparative clinical study	1,5,7,9,10,14,15,17
Shen et al., 2025 (20)	84	32.91 ± 4.42/32.74 ± 4.76	48 wk	Randomized comparative clinical study	1,2,5,7,9-15,17,20
Li et al., 2025 (21)	90	31.64 ± 5.18/33.36 ± 4.77	24 wk	Comparative clinical study	1,5,9,10,14,15,17,19
Ye, 2025 (22)	112	33.5 (28.0–43.5)/33.0 (28.0–41.0)	48 wk	Retrospective PSM comparative cohort	1-5,7-10,15,17
Zeng et al., 2025 (23)	80	42.39 ± 4.63/42.82 ± 4.66	6 mo	Randomized comparative clinical study	1,5,9-15,17
Pan, 2025 (24)	144	30.36 ± 12.12/30.79 ± 12.65*	24 wk; subset to 48 wk	Retrospective comparative cohort	1,2,5,7-15,17,18
van Vollenhoven et al., 2025 (25)	335	34.8 ± 9.8/35.1 ± 10.4	52 wk	Phase 3 randomized controlled trial	3,15-17
Huang et al., 2025 (26)	86	24.71 ± 9.43/32.75 ± 11.66	6 mo	Real-world comparative cohort	5,20
Chen et al., 2025c (27)	49	33 (25–45)	12 mo	Multicenter comparative LN cohort	6,15,17
Jin et al., 2025a (28)	201	31 (24–37)/32 (26–40)	52 wk	Retrospective comparative cohort	3,15,17
Fang et al., 2025 (29)	38	29.37 (24.59–35.32)*	52 wk	Retrospective single-arm cohort	3
Liu et al., 2025a (30)	12	30 (24–41)	12 mo	Retrospective single-arm LN cohort	1,2,7,9,10,15,18
Chen et al., 2025a (31)	146	32.04 ± 9.30/34.88 ± 10.20	12 mo	Retrospective comparative LN cohort	6,15
Feng et al., 2025 (32)	101	27 (19.8–35.5)/29 (21–38)	52 wk	Retrospective comparative cohort	3,4,15-17
Zhang et al., 2025b (33)	78	29.7 ± 7.3/30.5 ± 7.0	6 mo	Prospective comparative cohort	15,17,19
Chen et al., 2025b (34)	20	32.90 ± 7.19	12 mo	Prospective single-arm cohort	3,4,20
Jin et al., 2025b (35)	88	40.86 ± 12.96/40.82 ± 12.08	10.5 ± 3.78 mo	PSM comparative LN cohort	6
Liu et al., 2025b (36)	67	34.13 ± 10.25	52 wk	Single-center comparative LN cohort	3,6,15,16
Hu et al., 2025 (37)	61	35.43 ± 9.94	52 wk	Retrospective single-arm cohort	3,15,17
Gai et al., 2024 (38)	30	29.1 ± 8.9/34.5 ± 10.1/27.2 ± 3.9	6 mo	Three-group clinical observation	1,2,5,7-9,14,15,17,19
Xu, 2024 (39)	99	32.74 ± 13.11	6 mo	Multicenter retrospective comparative study	1-5,9,10,15,17
Li et al., 2024 (40)	102	34.28 ± 8.71	24 wk	Comparative clinical study	1,2,5,7,9,10,14,15,17
Niu et al., 2024 (41)	90	39.86 ± 5.91/39.07 ± 6.23/40.27 ± 6.89	NR	Three-group clinical observation	5,7,9,10,14,15,17
Wu et al., 2024 (42)	249	Mean 33.9	48 wk	Phase 2b randomized controlled trial	3,15-17
Ji et al., 2024 (43)	37	34.6 (28.8–46.1)	12 mo	Prospective single-arm cohort	3,15-17,20
Huang et al., 2024 (44)	30	30 (26–36.25)	48 wk	Retrospective single-arm LN cohort	3,6,15
Fan et al., 2024 (45)	87	30 (22–36)	24 wk	Single-center observational cohort	4,15
Cheng et al., 2024 (46)	22	35.76 ± 11.25	6 mo	Retrospective single-arm cohort	15,17,18
Lin et al., 2023 (47)	90	39.86 ± 6.72/40.27 ± 8.15	24 wk	Comparative LN clinical study	1,5-7,9-13,15,17,20
Qi, 2023 (48)	91	29.0 (20.0–34.0)/28.0 (21.0–34.3)/31.0 (21.5–40.0)	6 mo	Retrospective three-group cohort	1-3,5,7-10,15,17
Chen et al., 2023 (49)	20	38.1 (27.2–49.0)	Variable	Single-arm real-world observational study	3
Jin et al., 2023 (50)	72	32.72 ± 10.93	52 wk	Multicenter retrospective single-arm cohort	3,4,6,15-17
Jiang et al., 2020 (51)	22	32.3 ± 9.45/35.2 ± 16.34/41.4 ± 11.61/37.0 ± 13.3	48 wk	Retrospective analysis of a randomized trial subset	1,3,5,7-15,17,20

Outcome codes denote outcomes reported by each study; hyphenated ranges include all intervening codes: 1, SLEDAI/SLEDAI-2 K; 2, anti-dsDNA antibodies; 3, SRI-4; 4, LLDAS or DORIS remission; 5, non-standard clinical response/remission; 6, renal response (CRR/PERR/PRR/overall renal response or renal relapse); 7, 24-h proteinuria or UPCR; 8, renal function and related renal laboratory indices; 9, C3; 10, C4; 11, IgG; 12, IgA; 13, IgM; 14, glucocorticoid dose or dose reduction; 15, any adverse event/adverse reaction or adverse-event-related discontinuation; 16, serious adverse event; 17, infection; 18, hematologic outcomes; 19, gonadal or other organ-specific outcomes; 20, B-cell subsets, cytokines, tumor markers, or other mechanistic outcomes. NR, not reported; *age at disease onset.

**Table 2:** Evidence modules and quantitative coverage.

Evidence module	Number of models	Quantitative outcomes	Unique reports	Nominal module n	Model size (k; n)
Randomized efficacy and safety	4	SRI-4 response; any adverse event; serious adverse event; upper respiratory tract infection (48–52 weeks)	2	460	k = 2; *n* = 460
Conventional-treatment comparison	6	Clinical total effective response; SLEDAI/SLEDAI-2 K; C3/C4; any adverse event; infection (24–26 weeks)	7	608	k = 3–6; *n* = 264–528
Belimumab comparison	4	Any adverse event; infection; C3/C4 (telitacicept vs. belimumab)	5	546	k = 3–4; *n* = 244–434
Comparative adult LN	2	Complete renal response (CRR); primary efficacy renal response (PERR) (10–12 months)	2	128	k = 2; *n* = 128
Single-arm adult SLE efficacy	4	SRI-4 response (12, 24–26, and 48–52 weeks); LLDAS (24–26 weeks)	5	233	k = 2–4; *n* = 51–190
Single-arm LN and safety	4	Complete renal response (24–26 and 48–52 weeks); any adverse event; infection	6	303	k = 3–4; *n* = 76–205

Module-level sample sizes count each study's largest contributing denominator once within that module; modules overlap and must not be summed. Model-specific *n* values reflect the selected analytic population and may differ from the total sample size reported in Table 1 because of dose-arm selection, matching, or time-specific denominators.

**Table 3:** Complete random-effects meta-analysis results.

ID	Outcome and stratum	k	n	Estimate (95% CI)	I^2^（%）
A01	RCT efficacy: SRI-4 response (48–52 weeks)	2	460	RR 2.04 (1.66–2.50)	0.0
A02	RCT safety: any adverse event (48–52 weeks)	2	460	RR 1.09 (1.02–1.17)	0.0
A03	RCT safety: serious adverse event (48–52 weeks)	2	460	RR 0.68 (0.35–1.30)	37.6
A04	RCT safety: upper respiratory tract infection (48–52 weeks)	2	460	RR 1.31 (0.83–2.06)	69.2
A05	Conventional-comparator efficacy: clinical total effective response (24–26 weeks)	4	354	RR 1.19 (1.18–1.21)	0.0
A06	Conventional-comparator efficacy: SLEDAI/SLEDAI-2 K endpoint (24–26 weeks)	3	264	MD −1.81 (−3.02–0.60)	61.7
A07	Conventional-comparator biomarker: C3 endpoint (24–26 weeks)	6	528	MD 0.19 g/L (0.14–0.25)	11.4
A08	Conventional-comparator biomarker: C4 endpoint (24–26 weeks)	6	528	MD 0.06 g/L (0.03–0.10)	78.8
A09	Conventional-comparator safety: any adverse event (24–26 weeks)	6	464	RR 1.06 (0.37–3.03)	62.4
A10	Conventional-comparator safety: infection (24–26 weeks)	6	464	RR 1.06 (0.28–4.00)	25.6
A11	Belimumab-comparator safety: any adverse event (mixed 24–52 weeks)	4	434	RR 1.11 (0.84–1.49)	0.0
A12	Belimumab-comparator safety: infection (mixed 24–52 weeks)	4	434	RR 1.10 (0.75–1.60)	0.0
A13	Belimumab-comparator biomarker: C3 endpoint (24–26 weeks)	3	244	MD 0.14 g/L (0.07–0.21)	0.0
A14	Belimumab-comparator biomarker: C4 endpoint (24–26 weeks)	3	244	MD 0.04 g/L (−0.08–0.16)	87.0
A15	LN comparative efficacy: complete renal response (10–12 months)	2	128	RR 2.32 (1.30–4.16)	0.0
A16	LN comparative efficacy: primary efficacy renal response (10–12 months)	2	128	RR 1.67 (1.15–2.42)	9.9
A17	Single-arm efficacy: SRI-4 response (12 weeks)	3	119	Proportion 69.1% (40.0%–88.3%)	0.0
A18	Single-arm efficacy: SRI-4 response (24–26 weeks)	4	190	Proportion 76.2% (63.0%–85.7%)	0.0
A19	Single-arm efficacy: SRI-4 response (48–52 weeks)	2	51	Proportion 80.4% (67.2%–89.1%)	0.0
A20	Single-arm target attainment: LLDAS (24–26 weeks)	3	134	Proportion 44.8% (12.3%–82.4%)	78.1
A21	Single-arm LN efficacy: complete renal response (24–26 weeks)	3	102	Proportion 62.7% (29.4%–87.2%)	59.0
A22	Single-arm LN efficacy: complete renal response (48–52 weeks)	4	76	Proportion 66.8% (54.3%–77.4%)	0.0
A23	Single-arm safety: any adverse event (variable follow-up)	3	205	Proportion 15.7% (2.5%–57.4%)	79.2
A24	Single-arm safety: infection (variable follow-up)	3	205	Proportion 13.3% (1.8%–56.7%)	79.1

Proportions are shown as percentages; comparative estimates are RRs or MDs. A01-A24 are meta-analysis model identifiers used to link the results in Table 3, Table 5, and Figure 9; they do not denote individual studies.

Two reviewers agreed on item-level judgments for RoB 2, NOS, JBI, and GRADE. RoB 2 was applied to 13 outcome domains across five reports. The primary efficacy and safety outcomes in Wu et al., 2024 (42) and van Vollenhoven et al., 2025 (25) were all judged as having some concerns, mainly because of loss to follow-up and differences in post-discontinuation observation time. Nonstandard clinical response and some safety outcomes in Zhang et al., 2025a (19), Zeng et al., 2025 (23), and Li et al., 2024 (40) were judged at high risk of bias because of limited blinding, outcome measurement, and prespecified-protocol reporting. Most remaining objective laboratory or safety outcomes were judged as having some concerns. The nine comparative observational studies had NOS scores of 4–7. The eight uncontrolled cohorts had 7–8 Yes responses out of 10 on the JBI checklist. Key limitations were unclear consecutive or complete enrollment, declining follow-up denominators, and the absence of concurrent controls (Figure 2).

**Figure 2:**
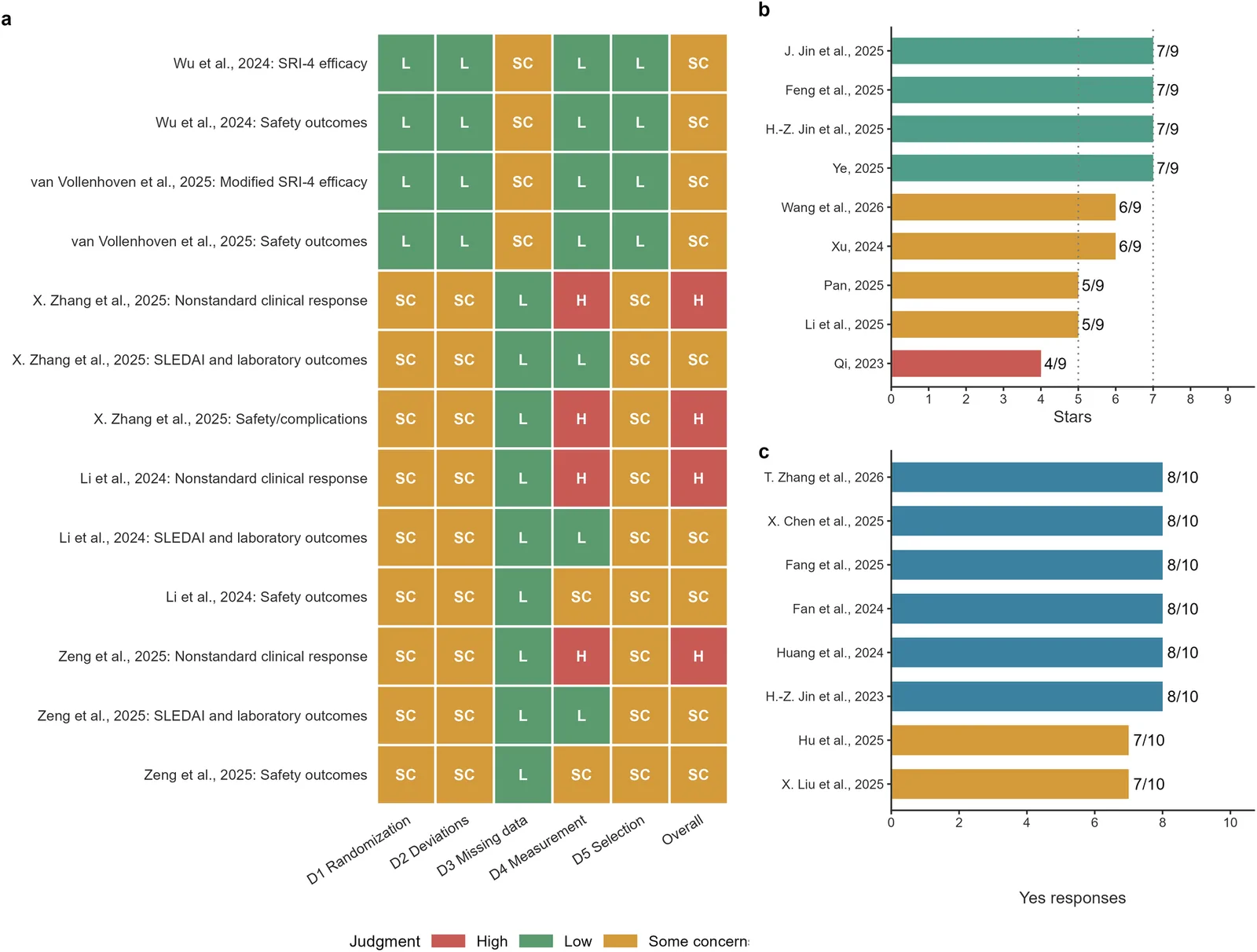
Risk of bias and methodological quality of studies contributing to the main metA,analysis models. **(a)** Outcome-specific RoB 2 judgments. **(b)** Newcastle-Ottawa Scale (NOS) item totals for comparative observational studies. **(c)** JBI case-series checklist responses for uncontrolled treatment cohorts. NOS totals and JBI responses are descriptive and are not validated low/high-risk thresholds.

### Efficacy and safety in randomized trials

Across two randomized trials, the analytic population comprised 460 participants receiving telitacicept 160 mg plus standard therapy or placebo plus standard therapy, with follow-up of 48–52 weeks^24,41^. Telitacicept increased the SRI-4 response rate (RR = 2.04, 95% CI 1.66–2.50; *P* < 0.001; I^2^ = 0.0%). The proportion of participants with any adverse event was slightly higher (RR = 1.09, 95% CI 1.02–1.17; *P* = 0.011; I^2^ = 0.0%).

No clear between-group differences were observed for serious adverse events (RR = 0.68, 95% CI 0.35–1.30; I^2^ = 37.6%) or upper respiratory tract infection (RR = 1.31, 95% CI 0.83–2.06; I^2^ = 69.2%) (Figure 3).

**Figure 3:**
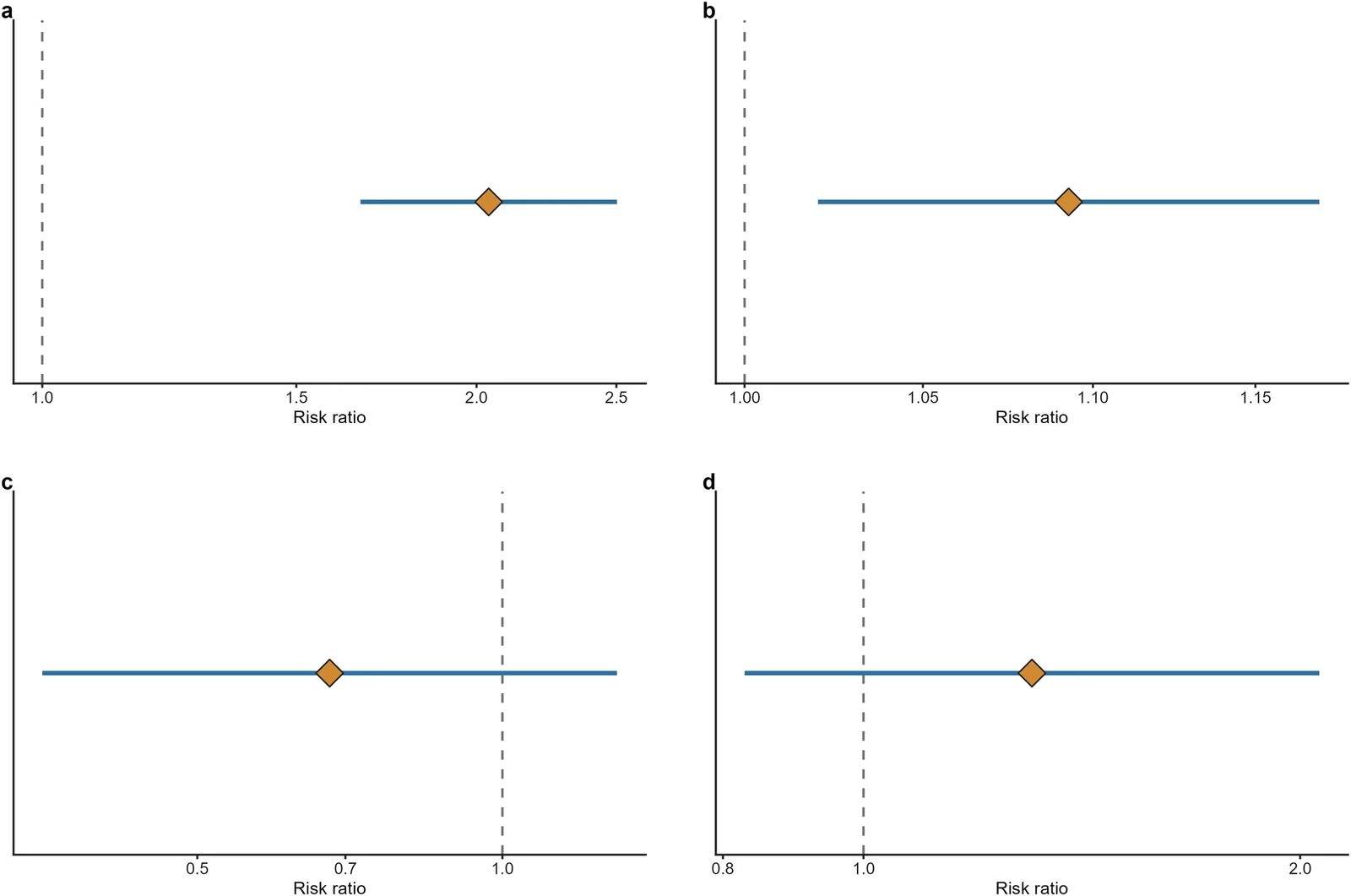
Randomized evidence at 48−52 weeks for telitacicept 160 mg plus standard therapy versus placebo plus standard therapy. **(a)** SRI-4 response; **(b)** any adverse event; **(c)** serious adverse event; and **(d)** upper respiratory tract infection. Points and horizontal lines represent random-effects estimates and 95% confidence intervals; each outcome was modeled separately.

### Comparison with conventional treatment regimens

At 24–26 weeks, the nonstandard clinical total effective response favored the telitacicept regimen (four studies, 354 participants; RR = 1.19, 95% CI 1.18–1.21; I^2^ = 0.0%), although definitions varied across studies and this endpoint is not equivalent to SRI-4 (19, 21, 23, 24, 39, 40, 48). Participants receiving telitacicept had lower SLEDAI or SLEDAI-2 K endpoint scores (three studies, 264 participants; MD = −1.81, 95% CI −3.02 to −0.60; I^2^ = 61.7%), higher C3 (six studies, 528 participants; MD = 0.19 g/L, 95% CI 0.14–0.25; I^2^ = 11.4%), and higher C4 (MD = 0.062 g/L, 95% CI 0.027–0.097; I^2^ = 78.8%). No clear differences were observed for any adverse event (RR = 1.06, 95% CI 0.37–3.03) or infection (RR = 1.06, 95% CI 0.28–4.00), and confidence intervals were wide (Figure 4).

**Figure 4:**
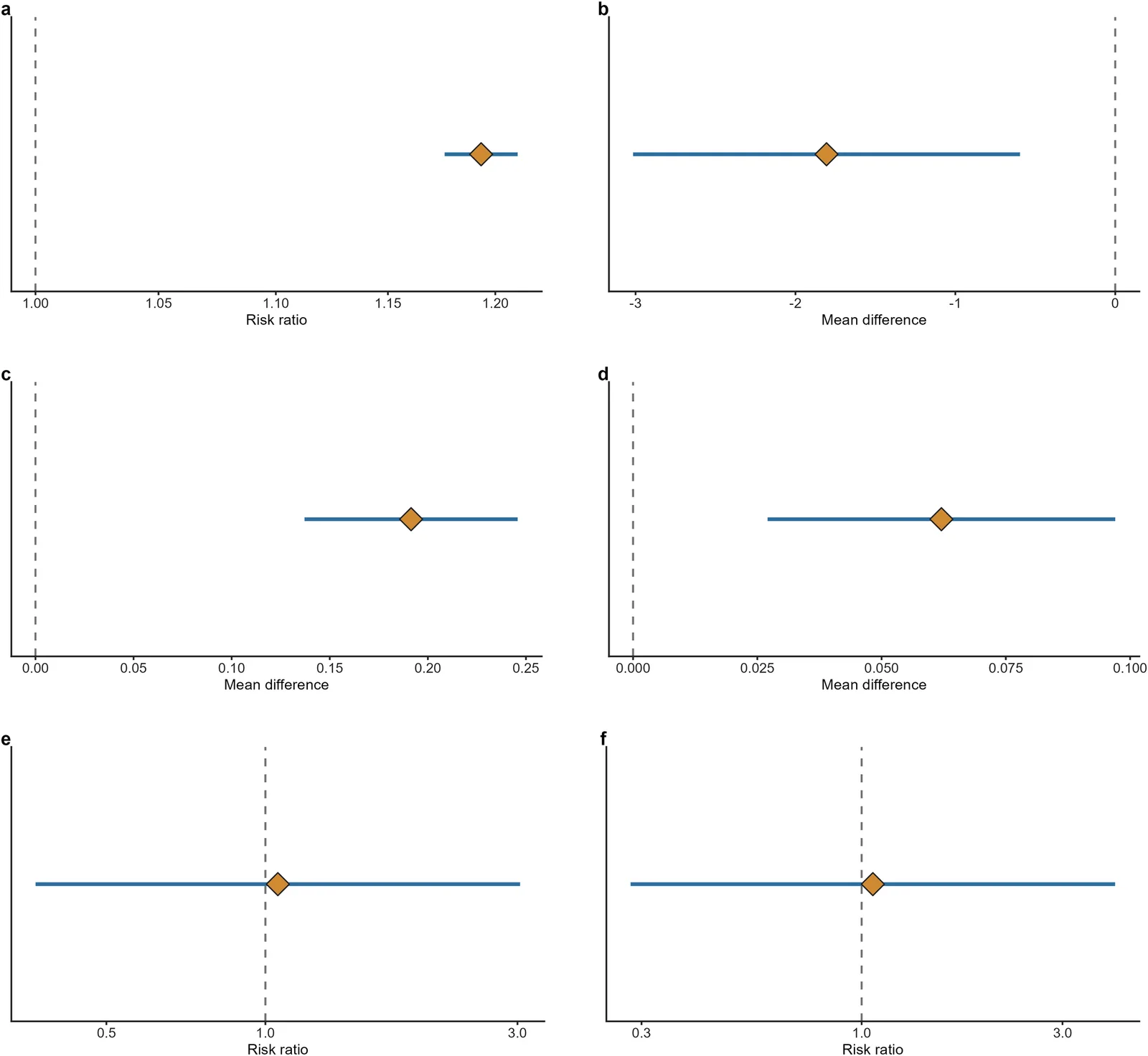
Telitacicept,containing regimens versus conventional regimens at 24-26 weeks. (**a)** Clinical total effective response, an exploratory nonstandard endpoint; **(b)** SLEDAI/SLEDAI-2 K endpoint; **(c)** C3; **(d)** C4; **(e)** any adverse event; and **(f)** infection. Outcomes were modeled separately, and no across-outcome summary estimate was calculated. Negative SLEDAI mean differences and positive C3/C4 mean differences favor telitacicept.

### Comparison with belimumab

Observational comparisons with belimumab showed no clear differences in any adverse event (four studies, 434 participants; RR = 1.11, 95% CI 0.84–1.49; I^2^ = 0.0%) or infection (RR = 1.10, 95% CI 0.75–1.60; I^2^ = 0.0%) (22, 28, 32, 39, 48). At 24–26 weeks, C3 was higher in the telitacicept group (three studies, 244 participants; MD = 0.14 g/L, 95% CI 0.07–0.21; I^2^ = 0.0%), whereas the C4 difference was uncertain (MD = 0.037 g/L, 95% CI −0.083–0.158; I^2^ = 87.0%). These unadjusted pooled endpoints cannot establish comparative efficacy between the two biologic agents (Figure 5).

**Figure 5:**
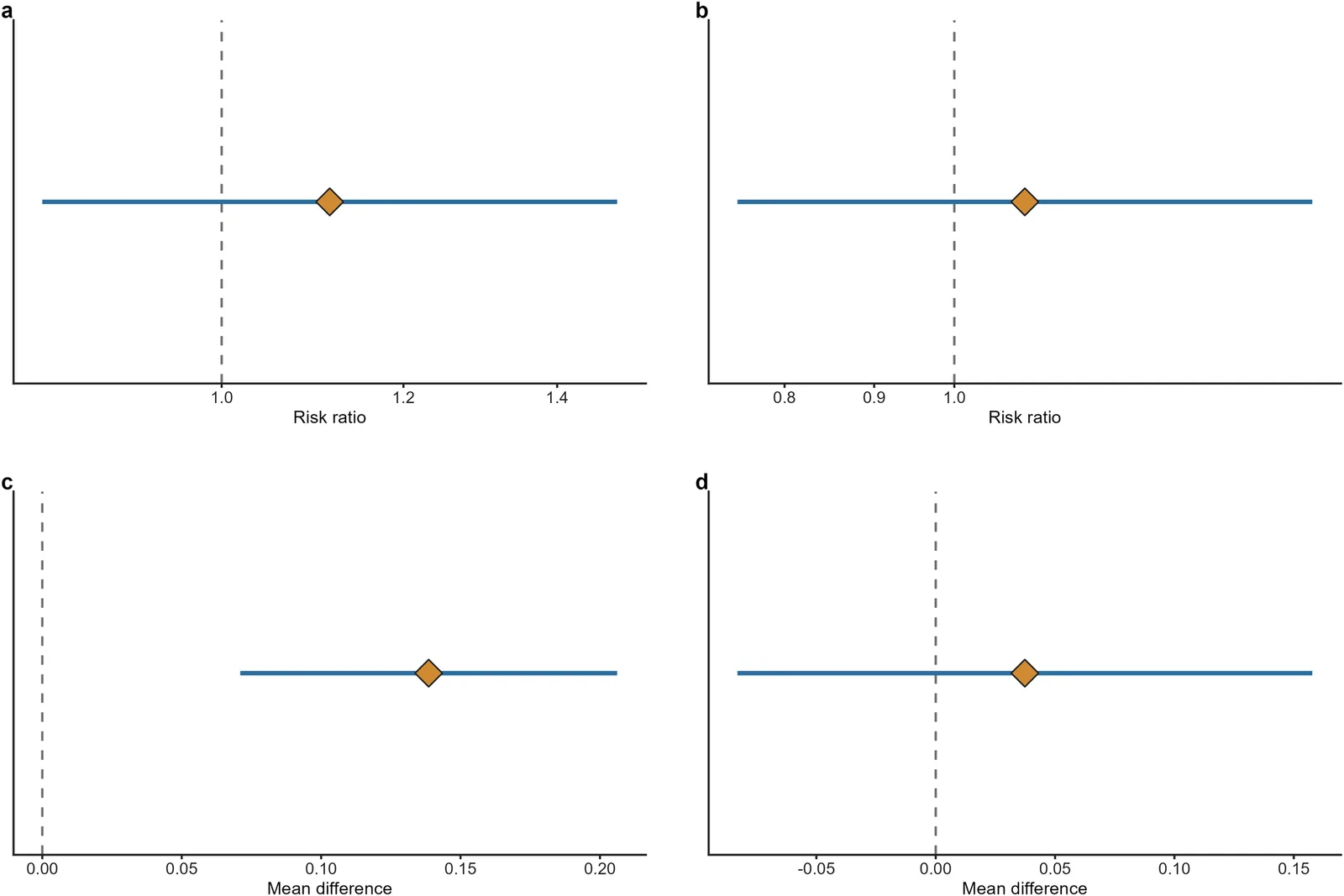
Observational comparisons of telitacicept with belimumab. **(a)** Any adverse event (mixed 24-52 weeks); **(b)** infection (mixed 24-52 weeks); **(c)** C3 at 24-26 weeks; and **(d)** C4 at 24-26 weeks. RRs <1 favor telitacicept, and positive MDs favor higher complement levels. In the sensitivity analysis, H.-Z. Jin et al., 2025 was replaced with Ye, 2025.

### Comparative evidence in adult lupus nephritis

Two observational comparative studies included 128 adults with LN and assessed renal response at 10–12 months (16, 35). Add-on telitacicept was associated with a higher CRR (RR = 2.32, 95% CI 1.30–4.16; *P* = 0.005; I^2^ = 0.0%) and a higher PERR (RR = 1.67, 95% CI 1.15–2.42; *P* = 0.007; I^2^ = 9.9%). Because both studies were nonrandomized, residual confounding and treatment selection may explain part of these associations (Figure 6). Key baseline characteristics, including renal pathology, proteinuria, glucocorticoid exposure, and concomitant immunosuppression, are presented in Table 4. Wang et al., 2026 reported no statistically significant baseline differences, although the groups differed numerically in proteinuria, biopsy availability, and pathological distribution. Jin et al., 2025b used propensity-score matching, but residual and unmeasured confounding remain possible. Accordingly, these findings indicate associations only and cannot establish a causal renal treatment effect or support treatment recommendations.

**Figure 6:**
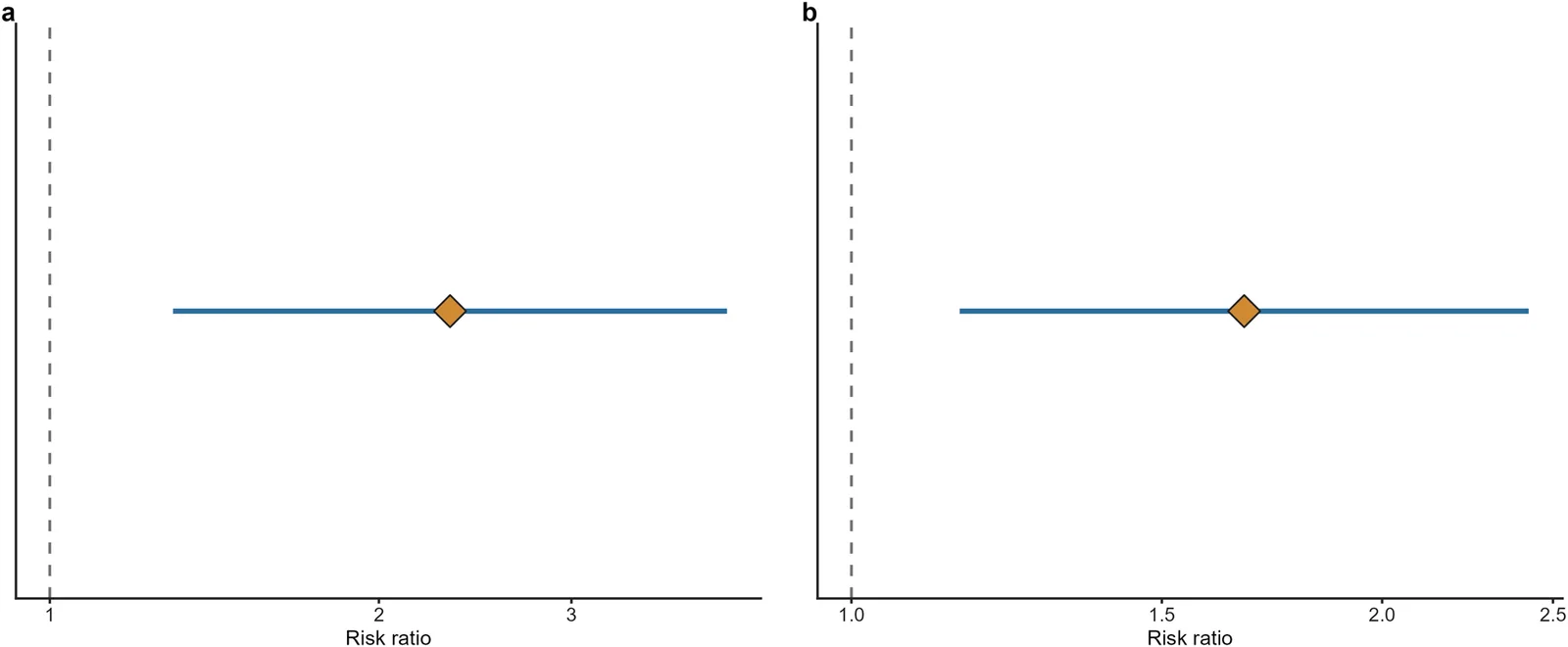
Comparative renal responses in adult lupus nephritis at 10-12 months for adD–on telitacicept versus standard therapy. **(a)** Complete renal response (CRR); and **(b)** primary efficacy renal response (PERR). The outcomes were modeled separately and not combined into a single overall effect.

**Table 4:** Baseline characteristics of the two comparative adult lupus nephritis cohorts.

Study	Analytic groups	Age, years	Baseline 24-h proteinuria (g/24 h)	Renal pathology	Baseline glucocorticoids and immunosuppression
Wang et al., 2026 (16)	Telitacicept + standard therapy vs. standard therapy 20 vs. 20	34.40 ± 14.96 vs. 31.15 ± 10.81	2.30 (1.73–3.30) vs. 3.47 (1.60–7.58)	II: 0 vs. 2; III/IV: 2 vs. 5; III/IV + V: 8 vs. 10; V: 3 vs. 2; no biopsy: 7 vs. 1	GC: 30.0 (30.0–42.5) vs. 40.0 (30.0–50.0) mg/day; MMF: 10 vs. 9; CTX: 2 vs. 5; TAC: 4 vs. 2; MMF + TAC: 4 vs. 3
Jin et al., 2025b (35)	PSM telitacicept + standard therapy vs. standard therapy 44 vs. 44	40.82 ± 12.08 vs. 40.86 ± 12.96	1.53 (0.06–9.72) vs. 1.21 (0.08–10.83)	III: 5 vs. 4; IV: 15 vs. 17; V: 7 vs. 5; III + V: 4 vs. 7; IV + V: 6 vs. 9; unknown: 7 vs. 2	GC: 42 vs. 44; HCQ: 33 vs. 33; MMF: 36 vs. 35; CTX: 1 vs. 3; TAC: 5 vs. 0; CsA: 3 vs. 3. Numerical baseline GC dose was not reliably extractable.

Values are telitacicept group versus control group unless stated otherwise. Data are mean+/-SD or median (range/IQR), as reported. PSM, propensity-score matching; GC, glucocorticoids; HCQ, hydroxychloroquine; MMF, mycophenolate mofetil; CTX, cyclophosphamide; TAC, tacrolimus; CsA, cyclosporine A.

### Single-arm efficacy in adult SLE

Across separately pooled time windows, SRI-4 proportions in telitacicept-treated cohorts were 69.1% at 12 weeks (three studies, 119 participants; 95% CI 40.0% to 88.3%), 76.2% at 24–26 weeks (four studies, 190 participants; 95% CI 63.0% to 85.7%), and 80.4% at 48–52 weeks (two studies, 51 participants; 95% CI 67.2% to 89.1%) (29, 34, 37, 45, 50). At 24–26 weeks, LLDAS attainment was 44.8% (three studies, 134 participants; 95% CI 12.3% to 82.4%; I^2^ = 78.1%). Some cohorts contributed multiple follow-up time points, but the studies and denominators were not identical across time windows. Therefore, time windows were not combined, and between-window differences were not interpreted as within-cohort efficacy trajectories (Figure 7).

**Figure 7:**
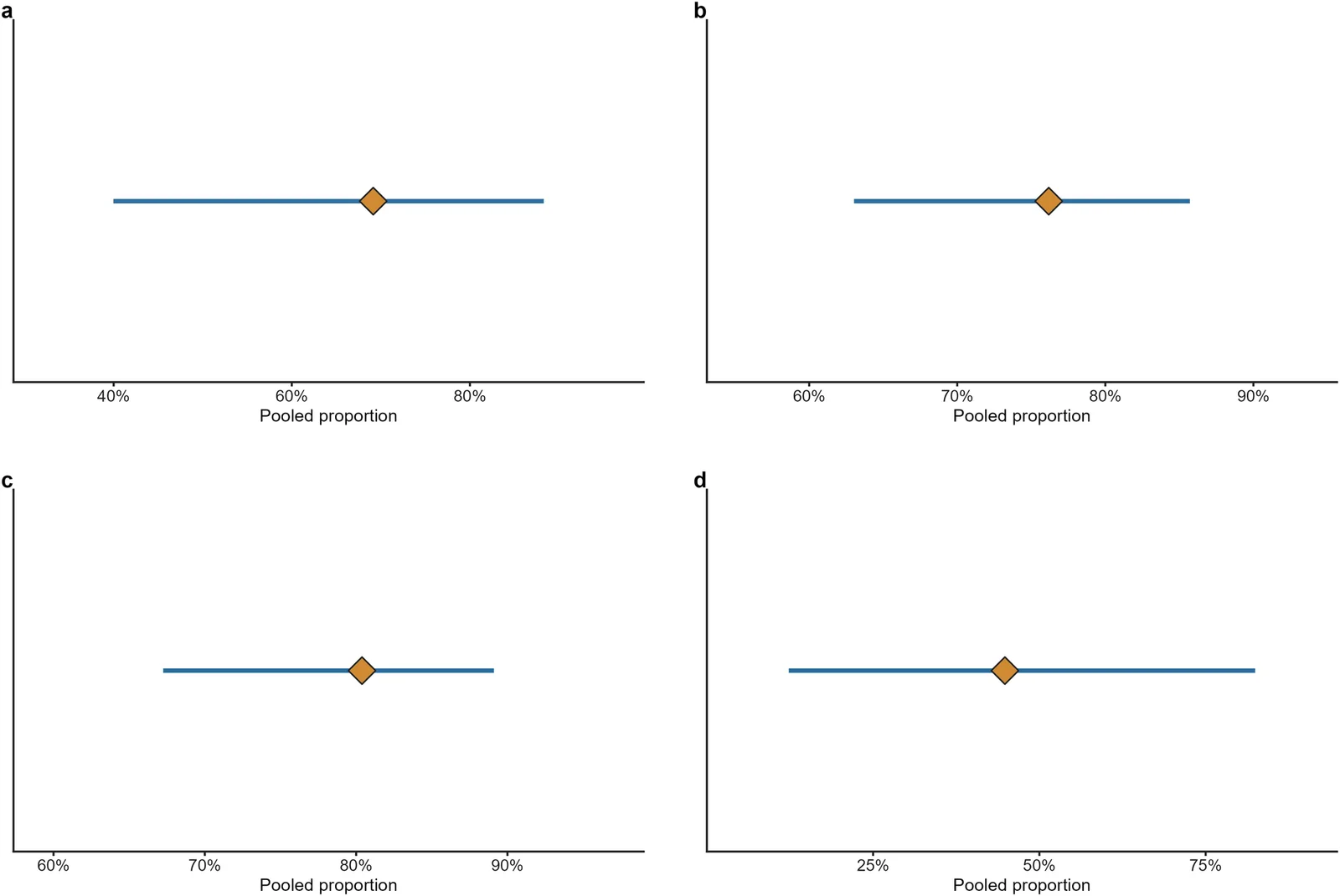
Single,Arm adult SLE efficacy. **(a)** SRI-4 response at 12 weeks; **(b)** SRI-4 response at 24-26 weeks; **(c)** SRI-4 response at 48-52 weeks; and **(d)** LLDAS attainment at 24-26 weeks. Proportions describe treated cohorts and do not represent comparative treatment effects; repeated time points were not pooled into a single overall proportion.

### Single-arm renal response and safety

Within adult single-arm LN cohorts, after Zhang et al., 2026 (18) was included as the primary-analysis report, the pooled CRR was 62.7% at 24–26 weeks (three studies, 102 participants; 95% CI 29.4% to 87.2%; I^2^ = 59.0%) (18, 36, 50) and 66.8% at 48–52 weeks (four studies, 76 participants; 95% CI 54.3% to 77.4%; I^2^ = 0.0%) (18, 36, 44, 50). Among three adult SLE cohorts with variable follow-up, the pooled proportion with any adverse event was 15.7% (205 participants; 95% CI 2.5% to 57.4%; I^2^ = 79.2%), and the pooled infection proportion was 13.3% (95% CI 1.8% to 56.7%; I^2^ = 79.1%). High heterogeneity and inconsistent event ascertainment limit interpretation of these safety proportions (Figure 8). Zhang et al., 2026 was retained as an independent 21-patient adult biopsy-proven LN cohort; however, possible partial center or calendar-period overlap with Jin et al., 2023 and Jin et al., 2025a could not be fully excluded from published information. Definitions ranged from treatment-emergent or treatment-related events to all events recorded during follow-up, and event ascertainment, follow-up duration, denominators, concomitant therapy, disease activity, and reporting practice differed across cohorts. A *post-hoc* descriptive check by reported follow-up showed any adverse event/infection proportions of 12/72 and 10/72 at 24 weeks in Pan, 2025, 19/72 and 17/72 with variable or unclear follow-up in Jin et al., 2023, and 4/61 and 3/61 at 52 weeks in Hu et al., 2025. Wei et al., 2026, evaluated only in sensitivity analysis because of inconsistent denominators, reported 32/94 and 24/94 at 52 weeks. Each follow-up stratum contained at most one primary-analysis study, precluding meaningful stratified pooling. These pooled safety proportions therefore describe a heterogeneous set of treated cohorts and must not be interpreted as stable overall incidence estimates or comparative safety effects.

**Figure 8:**
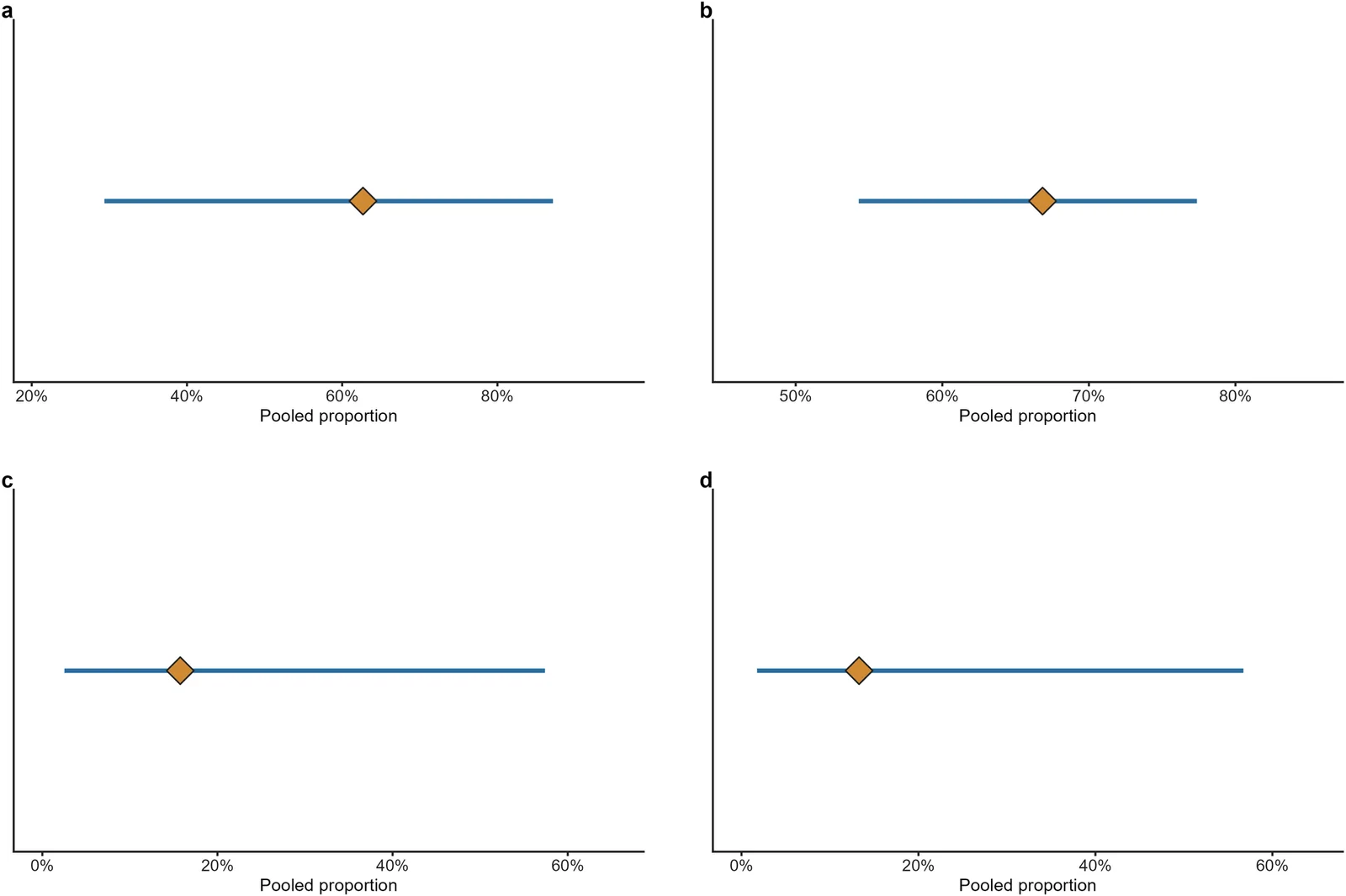
Single,Arm adult LN responses and adult SLE safety proportions. **(a)** Complete renal response at 24-26 weeks; **(b)** complete renal response at 48-52 weeks; **(c)** any adverse event; and **(d)** infection. T. Zhang et al., 2026 contributed to both renal-response models; time-specific renal denominators and variable safety follow-up were retained. The proportions describe treated cohorts and do not represent comparative effects.

### Sensitivity analyses and assessment of missing evidence

We performed 13 prespecified sensitivity analyses. Replacing the 160-mg group of the phase IIb trial with the 80-mg group yielded an SRI-4 RR of 2.06 (95% CI 1.68–2.53); replacing it with the 240-mg group yielded an RR of 2.10 (95% CI 1.72–2.58). Heterogeneity was I^2^ = 0.0% in both analyses. Adding the variable-follow-up outcome reported by Chen et al., 2023 to the 24–26-week single-arm SRI-4 model produced a pooled proportion of 76.5% (95% CI 67.3% to 83.8%). Other sensitivity analyses addressing study-design limitations, replacement of overlapping reports, and denominator restrictions did not change the direction of the main conclusions, although some confidence intervals remained wide. Because only one randomized trial contributed more than one telitacicept dose and its dose arms shared a placebo group, the dose substitutions were descriptive and did not permit a formal dose-response meta-analysis. Thus, stability of the directional SRI-4 signal across tested dose substitutions cannot establish a dose-response relationship, rank dose-specific benefit-risk, or identify an optimal dose. Ji et al., 2024 was not included in the primary models because of potential cohort overlap with Jin et al., 2025b. In overlap-related sensitivity analyses, replacing Jin et al., 2025a with Ye, 2025 in the belimumab-comparator safety models yielded RRs of 0.92 (95% CI 0.41–2.04) for any adverse event and 0.85 (95% CI 0.26–2.72) for infection. Adding Wei et al., 2026 to the single-arm safety models yielded pooled proportions of 19.8% (95% CI 6.2% to 48.0%; I^2^ = 85.4%) for any adverse event and 16.4% (95% CI 5.3% to 41.0%; I^2^ = 79.8%) for infection.

Each meta-analysis included only 2–6 studies. Therefore, funnel-plot asymmetry tests, trim-and-fill analyses, and related regression procedures were not conducted. The randomized SRI-4 outcome included only two trials from the same country and with the same funding source; the risk of missing evidence could not therefore be formally assessed. The supplemental registry search, limited to records available through 30 July 2026, identified no additional eligible study with publicly available full results, but it cannot exclude unpublished or incompletely reported evidence. We did not downgrade certainty solely because fewer than 10 studies were included. The risk of missing evidence was greater for nonrandomized and single-arm outcomes because small positive studies may be concentrated, outcome definitions were flexible, and prospective registration was incomplete.

### Certainty of evidence

No outcome was rated as high certainty by GRADE. SRI-4 response and any adverse event at 48–52 weeks were downgraded one level for risk of bias and were ultimately rated as moderate certainty. Serious adverse events were further downgraded to low certainty because events were sparse and the confidence interval crossed important benefit and harm.

Upper respiratory tract infection in randomized trials was rated as very low certainty because of inconsistency and imprecision. All models comparing telitacicept with conventional treatment or belimumab, as well as all single-arm descriptive models, were rated as very low certainty because of study design, risk of bias, imprecision, heterogeneity, indirectness, or selective reporting. Certainty for single-arm models describes proportions in treated cohorts only and cannot support causal treatment effects (Figure 9 and Table 5).

**Figure 9:**
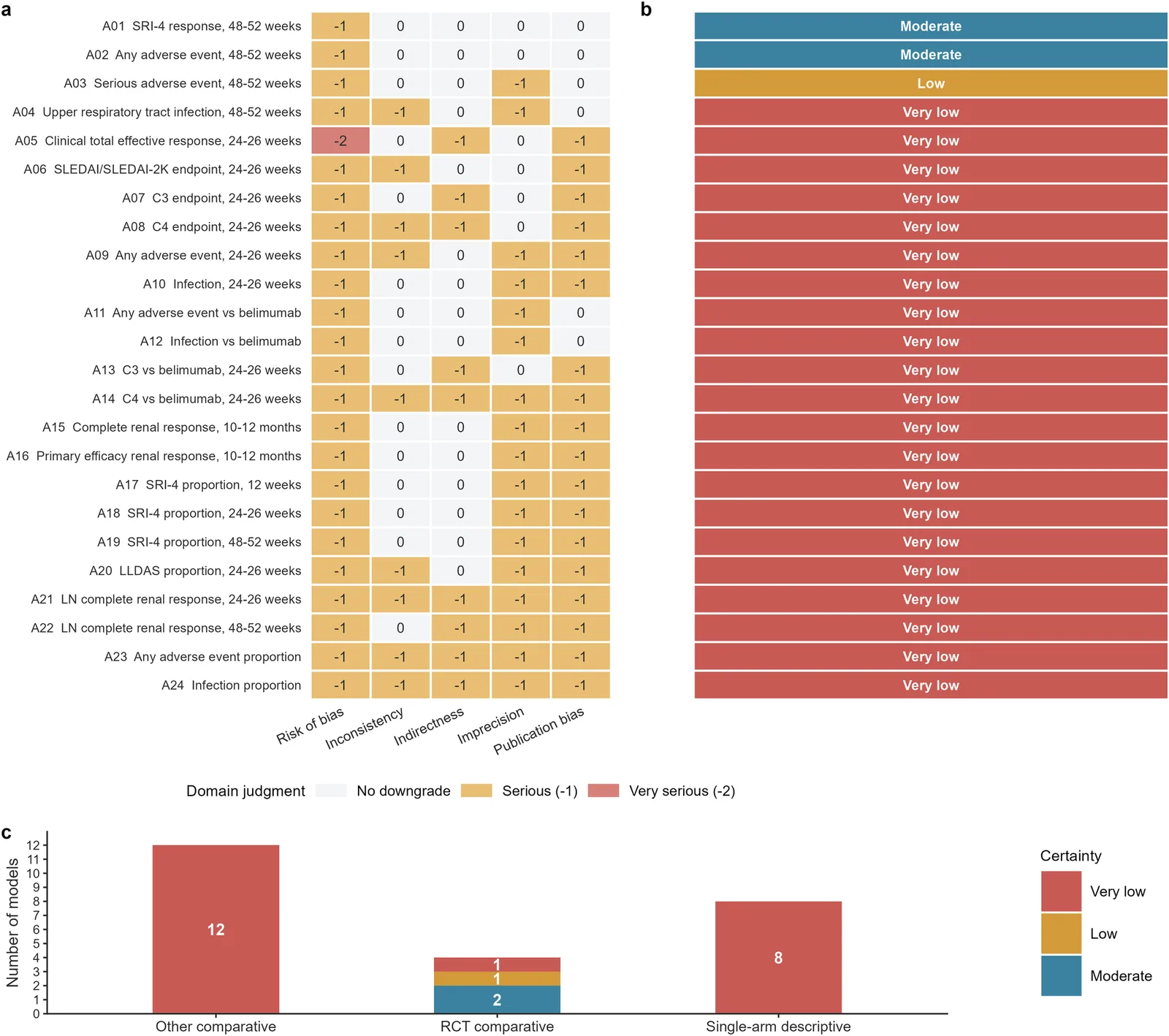
GRADE certainty of evidence across the 24 metA,analysis models. **(a)** Domain-level downgrades across the five GRADE domains (0, no downgrade; −1, serious; −2, very serious). **(b)** Final certainty assigned to each model. **(c)** Distribution of final certainty by evidence group (RCT comparative, other comparative, and single-arm descriptive). Model identifiers A01-A24 correspond to the results in Table 3. For single-arm models, certainty applies only to descriptive proportions among treated cohorts and does not support causal treatment effects.

**Table 5:** GRADE summary of findings for key outcomes.

ID	Outcome	k (n)	Effect (95% CI)	Absolute effect	Certainty	Interpretation
A01	SRI-4 response, 48–52 weeks	2 (460)	RR 2.04 (1.66–2.50)	+343 per 1,000 (+218 to +496)	Moderate	Telitacicept probably increases SRI-4 response at 48–52 weeks.
A02	Any adverse event, 48–52 weeks	2 (460)	RR 1.09 (1.02–1.17)	+78 per 1,000 (+17 to +143)	Moderate	Telitacicept probably slightly increases the proportion with any adverse event.
A03	Serious adverse event, 48–52 weeks	2 (460)	RR 0.68 (0.35–1.30)	-48 per 1,000 (−96 to +44)	Low	The effect on serious adverse events is uncertain.
A04	Upper respiratory tract infection, 48–52 weeks	2 (460)	RR 1.31 (0.83–2.06)	+85 per 1,000 (−48 to +294)	Very low	The effect on upper respiratory tract infection is very uncertain.
A06	SLEDAI/SLEDAI-2 K endpoint, 24–26 weeks	3 (264)	MD −1.81 (−3.02 to −0.60)	MD −1.81 points (−3.02 to −0.60)	Very low	The apparent SLEDAI reduction is very uncertain.
A15	Complete renal response, 10–12 months	2 (128)	RR 2.32 (1.30–4.16)	+227 per 1,000 (+51 to +543)	Very low	The comparative CRR association is very uncertain because evidence is observational.
A16	Primary efficacy renal response, 10–12 months	2 (128)	RR 1.67 (1.15–2.42)	+272 per 1,000 (+62 to +578)	Very low	The comparative PERR association is very uncertain because evidence is observational.

A01-A24 are meta-analysis model identifiers used consistently in Table 3, Table 4, and Figure 9; they do not denote individual studies. Certainty for single-arm models applies only to descriptive treated-cohort proportions and does not support causal treatment effects.

## Discussion

### Main findings and clinical positioning

The central conclusion is not that all evidence demonstrates efficacy for telitacicept. Rather, the strength of inference differs clearly by study design. In adults with active SLE represented by the two placebo-controlled randomized trials, telitacicept 160 mg plus standard therapy may increase SRI-4 response at 48–52 weeks and may slightly increase any adverse events. Together with low heterogeneity and moderate certainty of evidence, these findings provide a relatively robust basis for short-term clinical benefit with this regimen in the Chinese trial populations studied. They describe efficacy and safety signals within a defined population, dose, and follow-up period. They do not establish long-term disease control, prevention of organ damage, glucocorticoid tapering, remission or LLDAS achievement, or superiority to other biologic agents.

Presenting placebo-controlled randomized trials, other comparative studies, and single-arm cohorts separately, and appraising them according to their inferential strength, has direct clinical value. Randomized trials primarily address causal efficacy relative to placebo under trial conditions. Other comparative studies may provide association-based information in routine care but remain vulnerable to treatment selection and residual confounding. Single-arm studies only describe outcome proportions in treated cohorts. Therefore, the three evidence types are complementary rather than interchangeable. They should neither be pooled as a single overall effect nor used to extend the scope of randomized evidence using single-arm proportions or comparative associations.

### Interpretation and applicability of efficacy signals

The improvement in SRI-4 response in randomized trials was consistent with the conclusions of the two included trials. This consistency reinforces the internal coherence of the efficacy signal but does not establish that the observed clinical benefit is fully mediated by the proposed biological mechanism. The dose analysis was descriptive rather than a formal dose-response meta-analysis because the only multi-dose randomized trial used a shared placebo group and no independent dose-specific safety comparison was available across trials. When the 160-mg group of the phase IIb trial was replaced with the 80-mg or 240-mg group, the direction of the pooled SRI-4 effect did not change. This finding suggests that the main conclusion was not driven by a single dose group. However, these sensitivity analyses cannot establish a dose-response relationship or compare the benefits and risks of different doses.

Reductions in SLEDAI/SLEDAI-2 K and increases in C3 and C4 in conventional-treatment comparisons suggest potential improvement in disease activity measures and immunological biomarkers (52, 53). However, complement measures are biomarkers and cannot replace patient-important clinical outcomes (54). The clinical total effective response used in some studies is not a validated composite response measure. Its inconsistent definition means that it should not be treated as efficacy evidence as robust as SRI-4.

Similarly, differences in SLEDAI endpoint scores in non-placebo-controlled comparative studies may have been influenced by baseline imbalance, differences in concomitant therapy, lack of blinding, and incomplete reporting. Clinical interpretation should therefore be anchored in randomized SRI-4 evidence at 48–52 weeks. Other efficacy findings should primarily generate hypotheses for further validation rather than support definitive conclusions about comparative efficacy.

### Lupus nephritis and real-world evidence

The LN findings warrant attention because the pivotal systemic SLE randomized trials did not focus on organ-specific renal response as a primary endpoint. In this review, CRR and PERR favored telitacicept in two comparative cohorts. Following inclusion of Zhang et al., 2026, separately pooled single-arm analyses also reported renal response proportions in adult LN treatment cohorts across different time windows. These findings support prioritizing LN for confirmation in high-quality comparative studies. However, the evidence remains association-based or descriptive and cannot establish renal benefit or comparative advantage. Because all LN evidence was observational or single-arm and was graded as very low certainty, these findings are sufficient only to generate hypotheses and prioritize rigorous comparative trials; they do not support a clinical treatment recommendation.

Treatment selection in LN is influenced by baseline renal severity, prior and concomitant immunosuppressive therapy, refractory status, histopathological class, and clinician preference (55). Even when some studies used propensity-score methods, unmeasured confounding could explain part of the observed effect. Real-world findings are therefore better suited to defining research questions and optimizing trial design than to establishing treatment sequencing (56). Future LN studies should use consistent CRR and PERR definitions and prespecified follow-up windows. They should also fully report baseline renal characteristics, concomitant therapy, denominators lost to follow-up, and event counts to improve between-study comparability.

### Safety and benefit-risk considerations

For safety, pooled meta-analytic results showed an RR of 1.09 for any adverse event in randomized trials, indicating that the proportion may be slightly increased. Serious adverse events (RR = 0.68, 95% CI 0.35–1.30) and upper respiratory tract infection (RR = 1.31, 95% CI 0.83–2.06) showed no clear between-group differences. However, certainty of evidence for these outcomes was low and very low, respectively, and the findings should not be interpreted as showing comparable risk. Current safety evidence supports individualized monitoring for infection and immune-related risks during treatment. Long-term standardized studies are still needed to assess rare or delayed adverse reactions and the long-term benefit-risk balance. In single-arm cohorts, inconsistent adverse-event definitions, unequal follow-up, different denominators, concomitant therapy, and baseline disease activity are likely major contributors to the high heterogeneity (I^2^ approximately 79%). The corresponding pooled proportions describe heterogeneous cohorts rather than stable overall incidence estimates. Current evidence is dominated by short-term follow-up and remains insufficient to assess long-term serious adverse events, opportunistic infections, malignancies, and other delayed risks.

### Limitations and future directions

First, the evidence base remains limited. Each pooled model included only 2–6 studies, and all 39 included reports were conducted in China. Most non-placebo-controlled comparative studies and single-arm reports were small, single-center studies or lacked prospective registration. Randomized SRI-4 evidence also came from only two trials in the same country and with the same funding source. The results therefore should not be extrapolated directly to non-Chinese populations, in whom genetic background, baseline standard-of-care regimens, disease phenotype, access to biologics, and healthcare practice may differ. Consequently, between-study heterogeneity, geographic generalizability, small-study effects, and long-term safety could not be adequately assessed.

Second, outcome definitions and follow-up duration remained inconsistent at the systematic-review level, and some adjusted observational data lacked combinable variances or original event data. Because all models included fewer than 10 studies, funnel plots and regression-based asymmetry tests could not rule out the risk of missing evidence. Although we searched PubMed/MEDLINE, CENTRAL, CNKI, Wanfang, VIP, ClinicalTrials.gov, and WHO ICTRP, including ChiCTR records cross-checked through WHO ICTRP, Embase, Web of Science, and SinoMed were not searched because access was unavailable. Relevant reports or unpublished results may therefore still have been missed.

These limitations mean that current evidence supports continued evaluation of telitacicept 160 mg plus standard therapy in adults with active SLE who resemble the Chinese randomized-trial populations. It does not establish comparative efficacy or long-term safety beyond the boundaries of the available evidence. Future research should prioritize independent, multi-regional randomized trials that confirm SRI-4 efficacy at 48–52 weeks and extend follow-up. In LN, studies should use consistent CRR and PERR definitions and prespecified follow-up windows. They should also prespecify glucocorticoid tapering, LLDAS or remission, organ damage, patient-important outcomes, and long-term safety.

## Conclusion

Moderate-certainty evidence suggests that telitacicept 160 mg plus standard therapy may increase SRI-4 response at 48–52 weeks in Chinese adults with active SLE and may slightly increase any adverse events. Evidence for serious adverse events is of low certainty. Evidence for other safety outcomes, observational comparisons, and single-arm outcomes is of very low certainty and cannot establish comparative efficacy or causal effects. Current follow-up is insufficient to assess long-term serious adverse events, opportunistic infections, malignancies, and other delayed risks; therefore, a comprehensive long-term benefit-risk assessment is not yet possible. These findings support continued evaluation of telitacicept within the boundaries of the current evidence, while underscoring the need for independent multi-regional randomized trials, prospective LN studies, and more complete long-term safety data.

## Data Availability

The original contributions presented in the study are included in the article/Supplementary Material, further inquiries can be directed to the corresponding authors.
